# Asbestos exposure determined 357 days after death through autopsy: a report of a multidisciplinary approach

**DOI:** 10.1007/s12024-024-00838-z

**Published:** 2024-05-28

**Authors:** Giuseppe Davide Albano, Vito Rodolico, Simone Di Franco, Giuseppe Lo Re, Mauro Midiri, Ginevra Malta, Emanuele Cannizzaro, Antonina Argo, Stefania Zerbo

**Affiliations:** 1 Institute of Legal Medicine, Department of Health Promotion, Mother and Child Care, Internal Medicine and Medical Specialties, University of Palermo, Via del Vespro 129, Palermo, 90100 Italy; 2 Pathological Anatomy Department, Department of Health Promotion, Mother and Child Care, Internal Medicine and Medical Specialties, University of Palermo, Palermo, Italy; 3 Radiology Department, Department of Biomedicine, Neuroscience and Advanced Diagnostics, University of Palermo, Palermo, Italy

**Keywords:** Asbestosis, Autopsy, Exhumation, Scanning electron microscopy, Asbestos fibers, Multidisciplinary approach, Post-mortem CT (PMCT)

## Abstract

Asbestosis is an interstitial lung disease caused by the inhalation of asbestos fibers and poses a significant risk to individuals working in construction, shipping, mining, and related industries. In a forensic context, postmortem investigations are crucial for accurate diagnosis, for which the gold standard is the histopathological examination. This case report describes the autopsy and related investigations conducted on an 84-year-old man, nearly one year (357 days) after his death. After a post-mortem CT scan, an autoptic investigation was performed, followed by histopathological, immunohistochemical, and scanning electron microscopy examinations. The integration of the evidence from these examinations with previously available personal and clinical information conclusively confirmed the diagnosis of asbestosis. We demonstrated the efficacy and reliability of our diagnostic protocol in detecting asbestosis and asbestos fibers and excluding mesothelioma even in decomposed tissues. According to our findings autopsy remains the diagnostic gold standard in cases of suspected asbestosis within a forensic context, even 1 year after death, therefore it is always highly recommended, even in cases where the body has decomposed.

## Introduction

Asbestosis is an interstitial lung disease caused by inhaling asbestos fibers, which have historically been used in construction and similar fields due to their high electrical and thermal resistance and low cost. The symptoms of asbestosis include difficulty breathing, persistent coughing, wheezing and, in severe cases, respiratory failure [1]. These symptoms along with pleural effusions, pleural plaques, diffuse pleural fibrosis/thickening, and round atelectasis, are characteristic of non-neoplastic asbestos-related conditions. Such conditions are distinct from neoplastic asbestos-related conditions, such as malignant mesothelioma and lung carcinoma [2]. According to the *National Mesothelioma Register* (*ReNaM*), more than 31.000 cases of neoplastic asbestos-related conditions were registered in Italy from 1993 to 2018 [3]. Once the danger of asbestos fibers pose to public health was discovered, all products containing asbestos were banned in terms of production and marketing [4]. The assessment of a causal relationship between asbestos exposure and diseases or death is an essential public health, judiciary, and social insurance issue, especially in the case of an occupational disease. In this regard, a postmortem examination becomes necessary in deaths that arise for inquests [5] or are alleged to be a result of negligence [6]. Indeed, the value of autopsy in cases of death resulting from occupational diseases is also crucial, for judicial, insurance, and litigation issues [7, 8]. The diagnostic gold standard in such circumstances is histopathological and the use of scanning electron microscopy examinations, which are used to demonstrate the presence of interstitial fibrosis and two or more asbestos fibers per square centimeter of a 5-*µ*m-thick lung section, as stated by the Helsinki criteria [9–11]. Autopsies on decomposed bodies usually present numerous problems, as changes due to decomposition are known are known to give rise to misinterpretation [12]. Still, recent studies show that it’s helpful to proceed to the exhumation of corpses and to the autopsy for investigating asbestos exposure in corpses for up to 16–18 months after death [13, 14].

In this case report, we describe the application of a multidisciplinary approach in a judiciary autopsy case of a 84year-old man, almost a year (357 days) after his death, which led to the diagnosis of asbestosis.

## Case report

**History** A man was found dead at home at the age of 84 years. He worked for over 30 years in a shipyard, where he was exposed daily to asbestos materials. In the early 1980s, it was found that he could not continue this job. He was hospitalized twice due to multiple episodes of pneumonia, weight loss, vomiting, and diarrhea, and a few months after the second hospitalization, he died. The death occurred at home, it was unwitnessed and during the last months of life he continued to lose weight. He also suffered from hyperthyroidism, atherosclerosis, and hypertensive cardiopathy associated with decompensated heart failure. No signs of pulmonary failure or dyspnea were observed during life. thorax CT scan was performed 1 month before his death, showing lung interstitial fibrosis and pleural thickening.

At 357 days after his death, an autopsy was requested by the judicial authority to assess the presence of asbestos-related diseases due to occupational exposure.

### Post-mortem CT (PMCT)

**Fig. 1:**
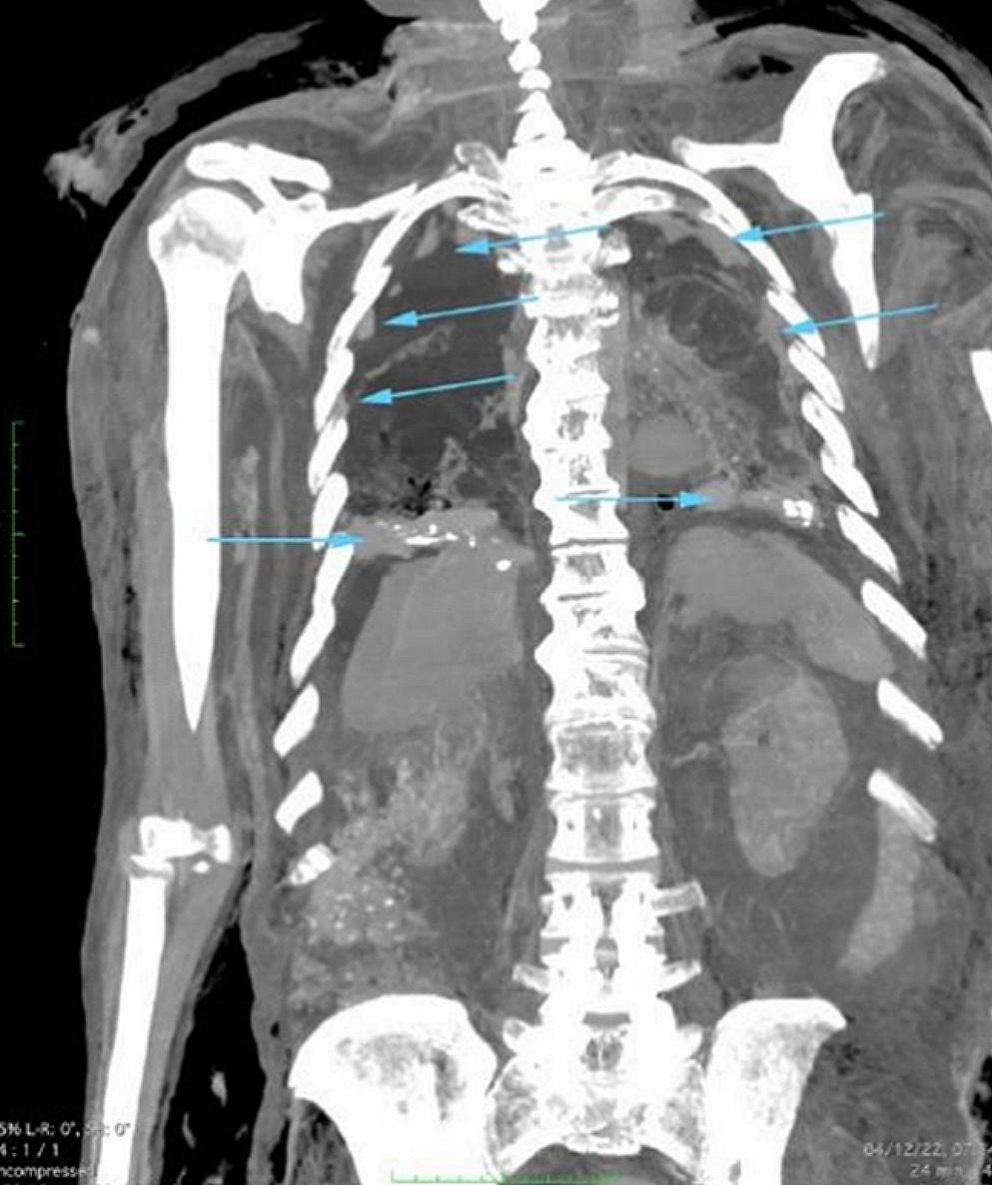
Coronal reconstruction (bone window), showingevidence of various pleural plaques, with signs of calcification (*blue arrows*)

**Fig. 2:**
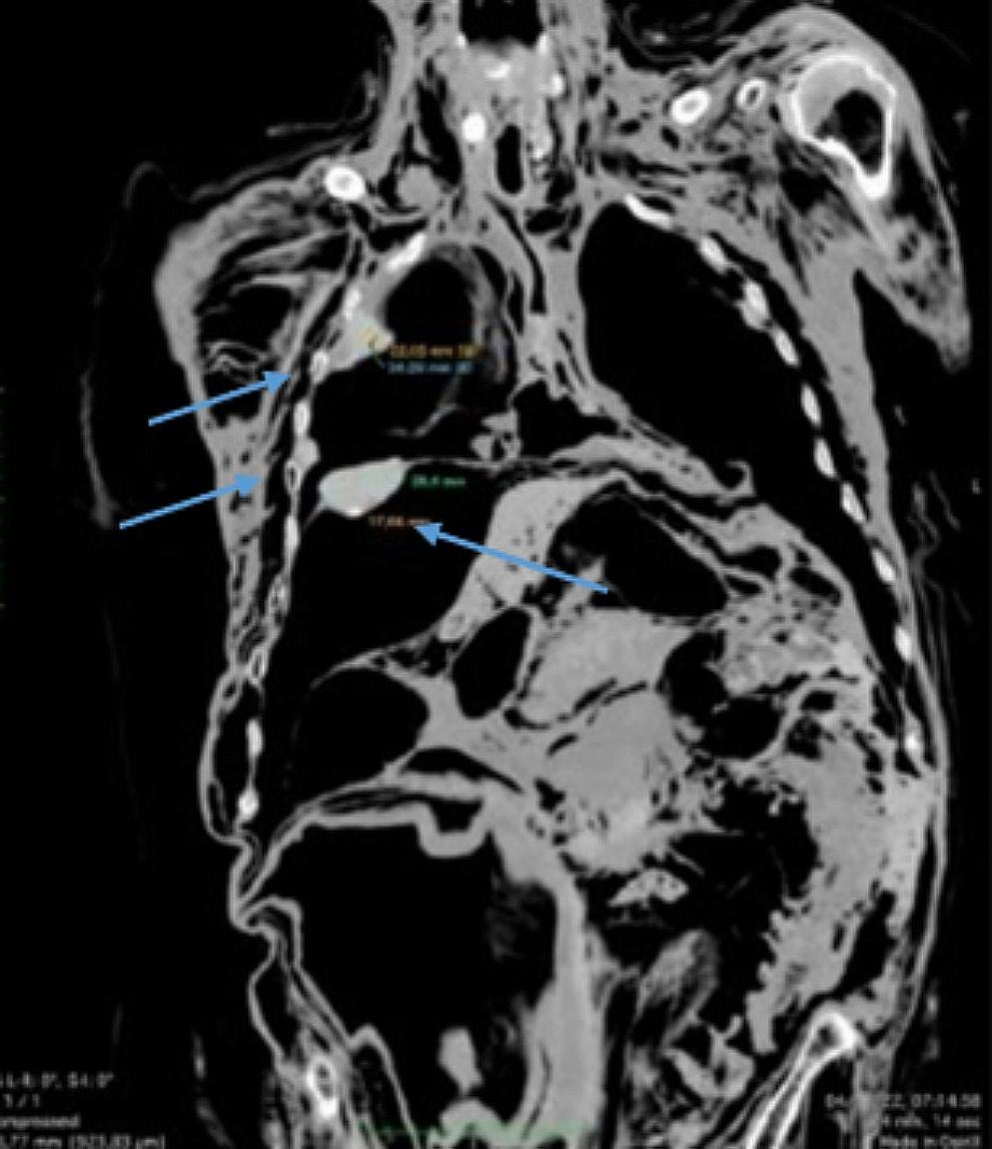
Coronal reconstruction (mediastinal window), showing evidence of various pleural plaques (parietal and diaphragmatic pleura) (*blue arrows*)

After the man’s body enveloped in a bag was subjected to a non-contrast whole-body-scan, PMCT examination was performed the day after the exhumation meaning 357 days after his death,, using 128-slices scanner (Somatom Definition AS®, Siemens Healthcare Erlangen, Germany). Images were reviewed using a dedicated workstation (Singovia Siemens Healthcare® Erlangen, Germany), with multiplanar reformatting (MPR), maximum-intensity-projection (MIP) and volume rendering (VR) reconstruction using soft-tissue, bone and lung windows. PMCT whole-body scans revealed multiple voluminous nodulations and thickenings of the entire pleura, partially calcified, especially in the diaphragmatic zones (Figs. 1 and 2). The pulmonary parenchyma was severely damaged due to putrefactive degeneration, and a significant amount of fluids was localized in the pleural space.

## Autopsy

An autopsy was performed almost a year (357 days) after the man’s death.

**Fig. 3:**
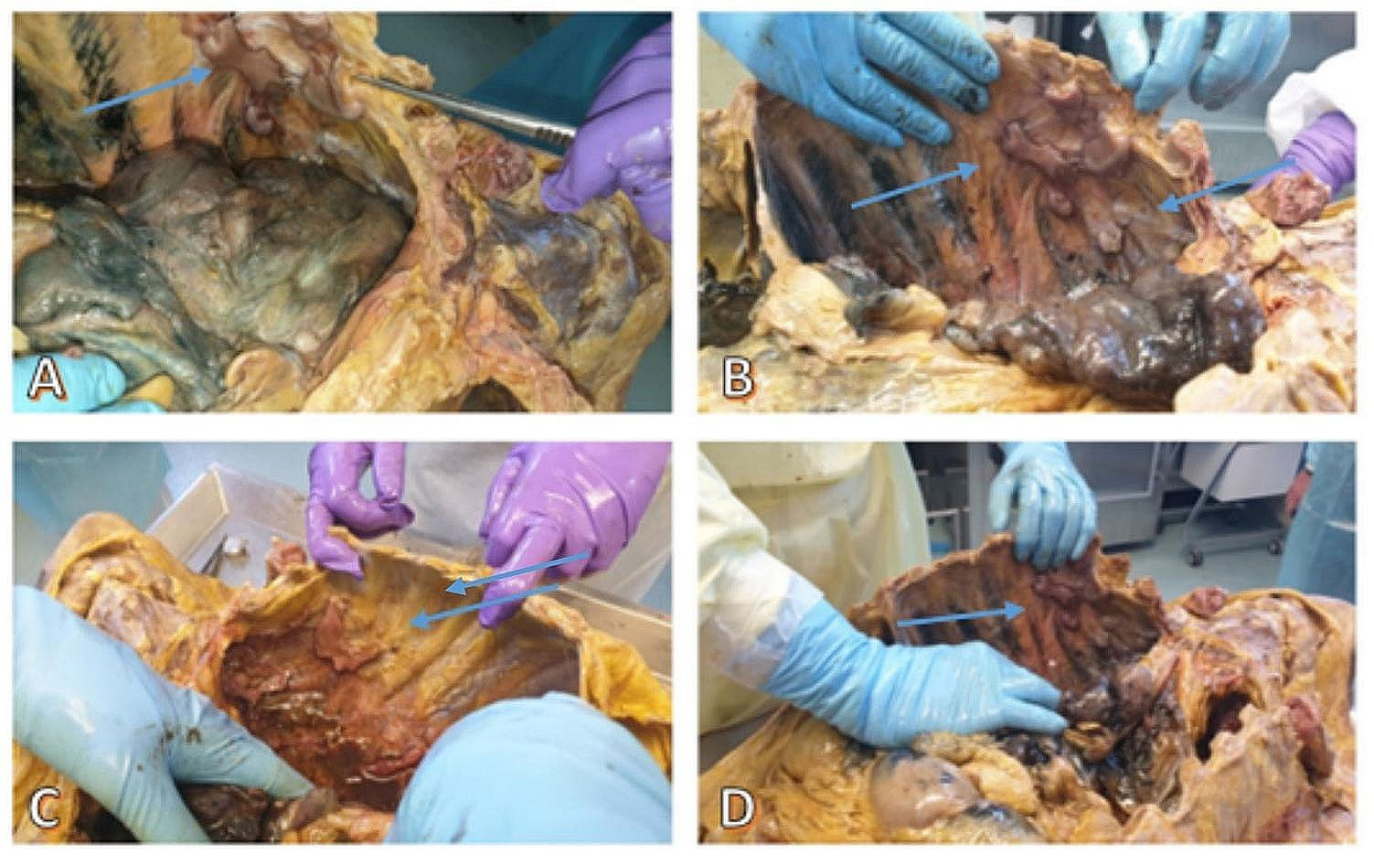
Macroscopic examination of the ribcage: **A, B, C, D**: evidence of multiple fibrotic adhesions along the right and left ribs, all covered in vegetative neoformations (*blue arrows*)

An external examination showed conspicuous putrefactive phenomena. The corpse was oily and soggy, widely covered in brownish and yellowish liquid due to the high post-mortem interval. The body height was 175 cm. An internal thoracic examination showed the presence of putrefactive liquid in pleural spaces and multiple, strong, extensive and diffuse fibrotic adhesions involving both lungs, especially on the diaphragmatic surface. In the right cavity, we found pleural-costal adhesions on the lateral posterior side of ribs I, II, III, IV and V and pleural diaphragmatic adhesions along the lung base, all covered in vegetative neoformations. In the left cavity, we found pleural-costal adhesions between the paravertebral line and the scapular line (from rib I to V), up to the axillary line (from rib III to V), all covered in vegetative neoformations (Fig. 3).

The thoracic organ block (Ghon’s technique) was removed together with the diaphragm due to significant basal and bilateral adhesions. No other significant findings were observed during autopsy.

**Fig. 4:**
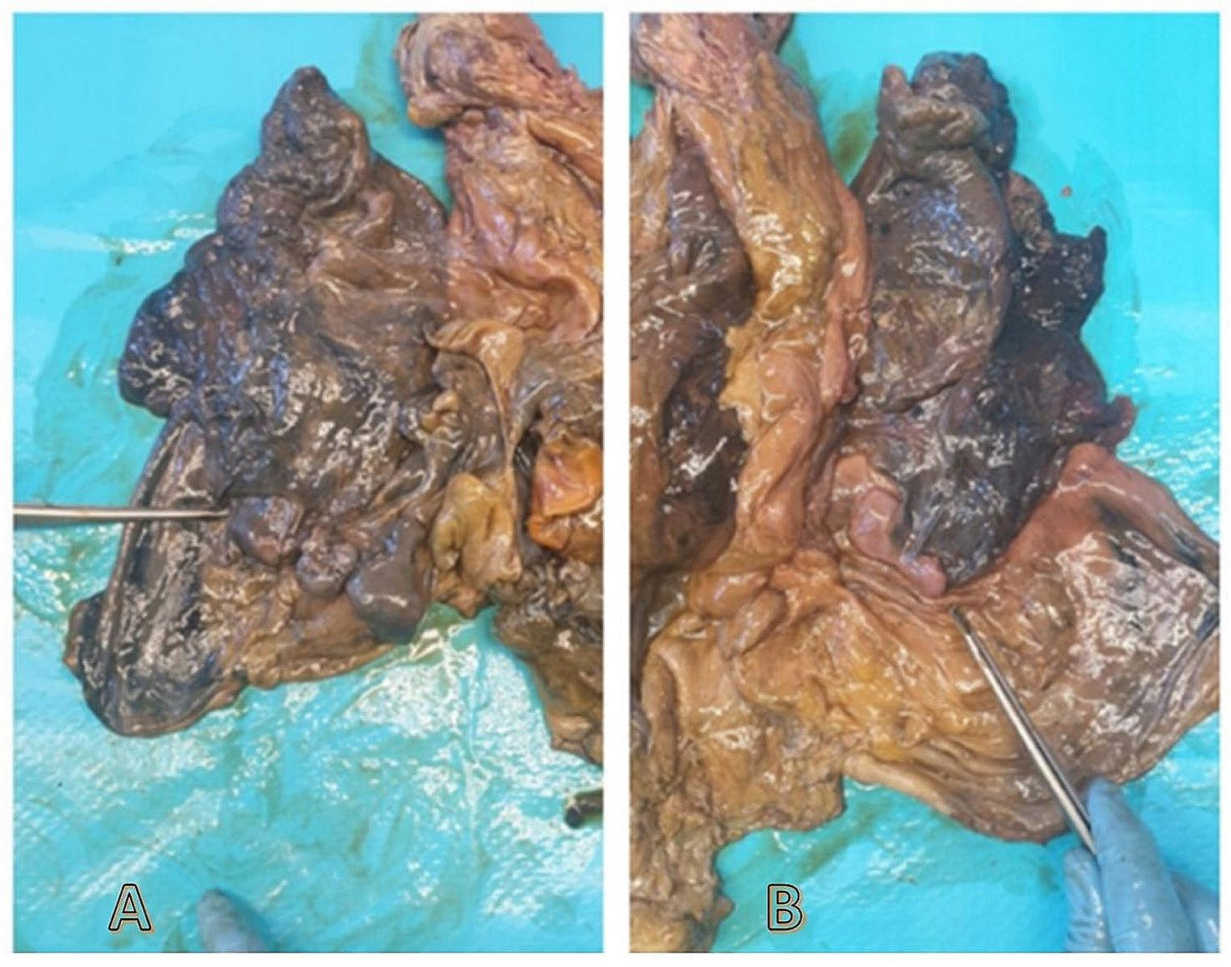
Macroscopic examination of thoracic organs bloc district: **A, B** evidence of pleural plaques (*forceps*) at both lungs’ bases

Thick and irregular neoformations were observed on the diaphragmatic sides of both lungs’ after the removal of thoracic organ block (A for the left lung, B for the right lung) (Fig. 4).

**Fig. 5:**
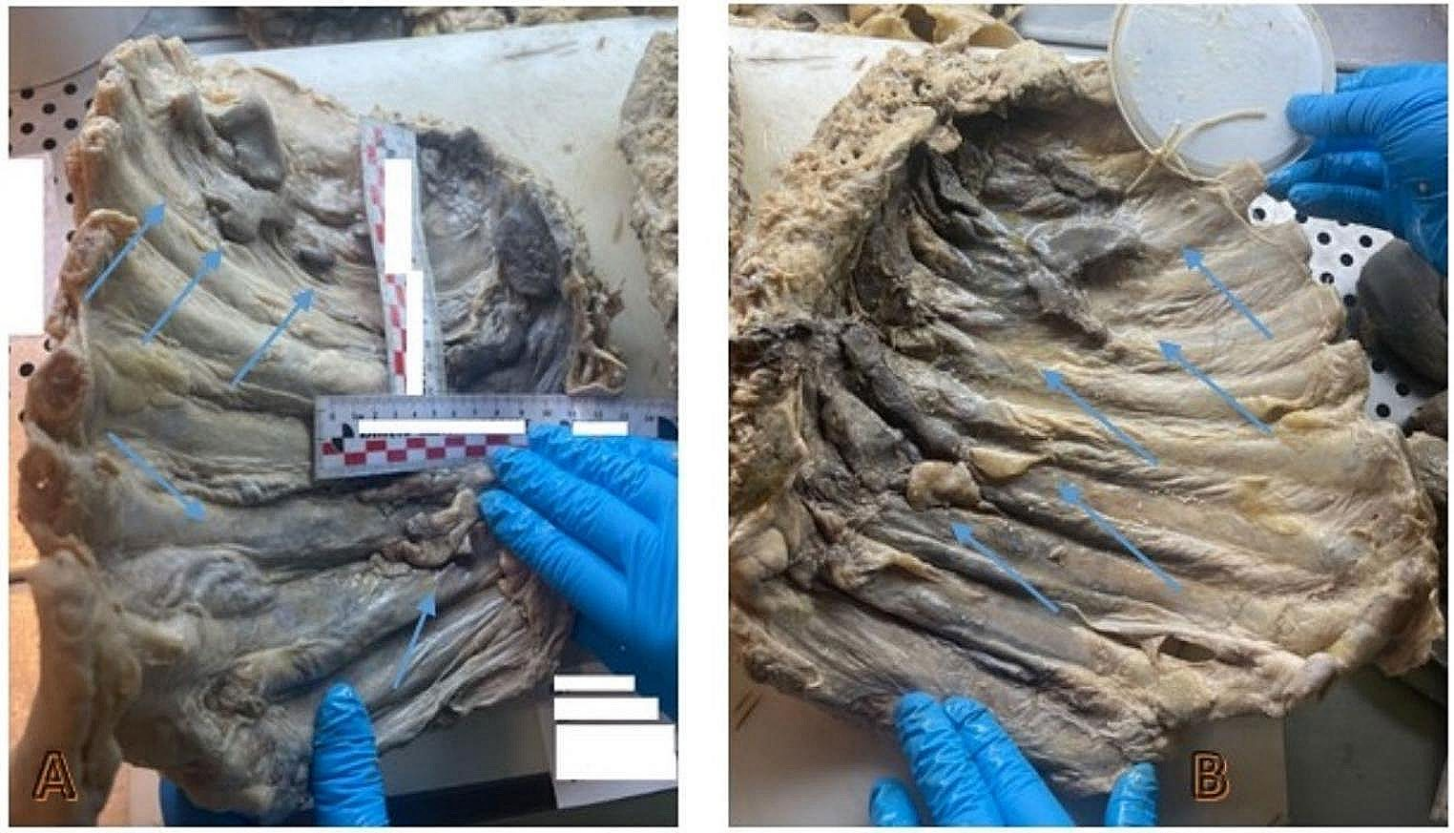
Macroscopic examination of left (**A**) and right (**B**) hemicostates after formalin fixation: evidence of multiple, gray and irregular vegetative neoformations strictly adhered to the hemicostates (pleural plaques)

The evidence of these fibrotic adhesions was confirmed during macroscopic examination in both ribcages after fixation (10% buffered formalin solution, 43 days after autopsy), which were especially prominent along the paravertebral line after ribcage isolation (Fig. 5).

### Histopathology

**Fig. 6:**
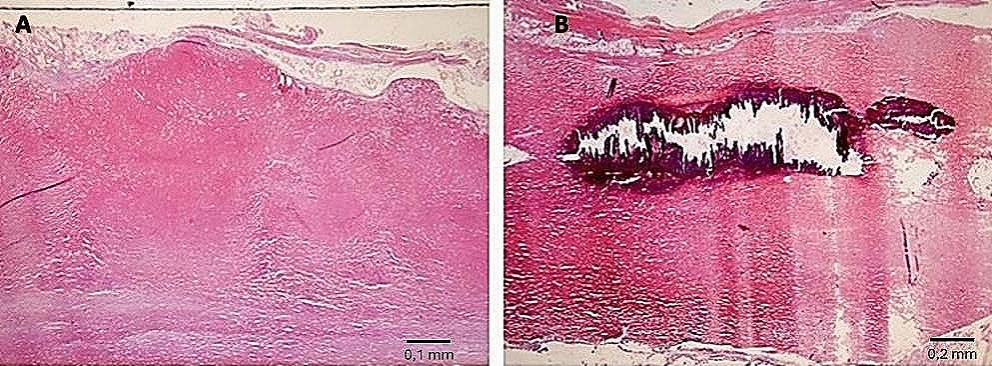
Parietal pleura (**A**, H&E, 10X; **B**, H&E, 20X): fibrotic plaques with acellular hyalin collagen fibers in a reticular pattern, including areas with calcium salt deposits

**Fig. 7:**
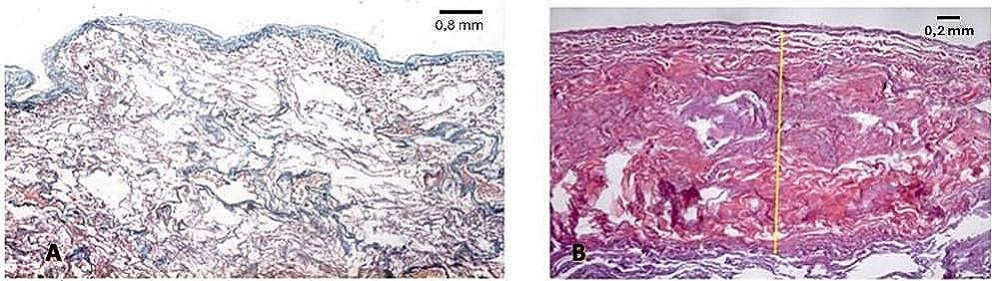
Visceral pleura: **A** thickening caused by fibrotic phenomena (Masson’s trichrome, 2.5X); **B** pleural thickness = 1.5 mm (*yellow line*) (H&E, 20X)

Pathological features were estimated using histological sections stained with hematoxylin–eosin, (H&E), and trichrome stains (Masson, Van Gieson). Histological examination of parietal pleural samples showed acellular hyalin collagen fibers in a reticular pattern, including areas with calcium salts deposit (Fig. 6). Visceral pleural samples showed multiple areas with fibrotic thickening (Fig. 7).

**Fig. 8:**
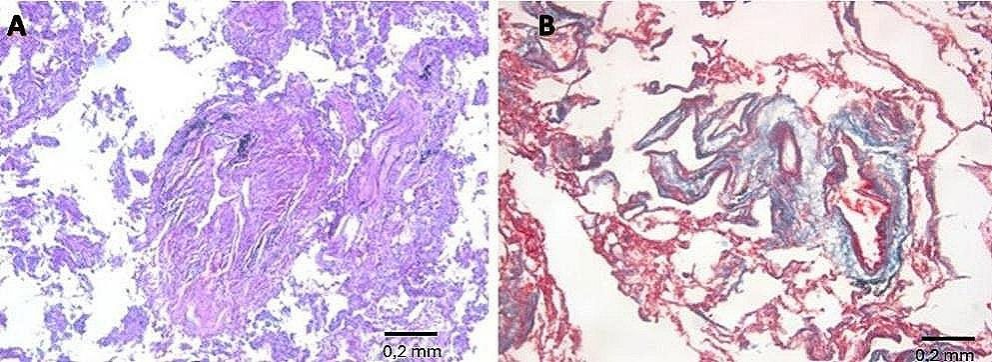
**A** Fibrosis of respiratory bronchiole walls (H&E, 20X), extending (**B**) to alveolar ducts and adjacent alveoli (Masson trichrome, 20X)

**Fig. 9:**
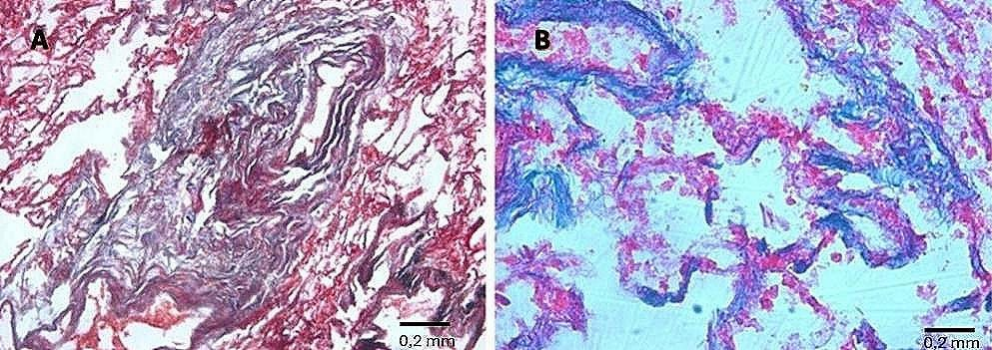
**A** Fibrosis of respiratory bronchiole wall (Van Gieson trichrome, 20X); **B** fibrosis of alveolar ducts and alveoli walls (Masson trichrome, 20X)

**Fig. 10:**
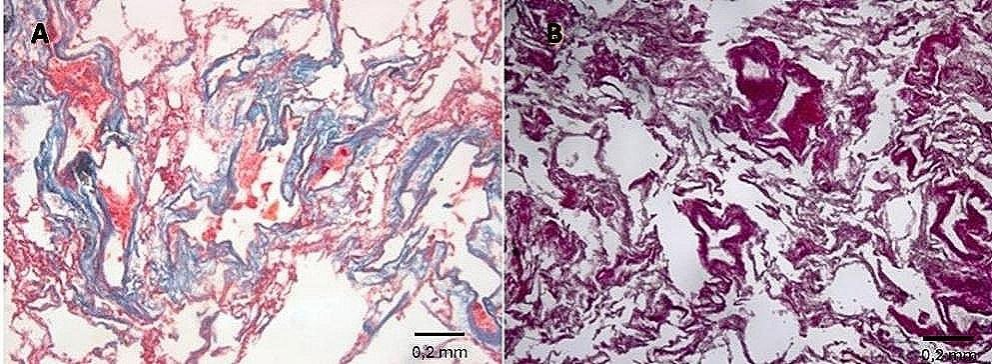
**A** Fibrosis of alveolar ducts and alveoli walls (Masson trichrome, 20X); **B** fibrotic thickening of the inter-alveolar septa between two or more contiguous respiratory bronchioles (Van Gieson’ trichrome, 20X)

Histological examination of pulmonary samples showed fibrosis of respiratory bronchiole walls, extending to alveolar ducts and adjacent alveoli (Fig. 8). There was fibrotic thickening of the inter-alveolar septa between two or more contiguous respiratory bronchioles, with higher representation in subpleural districts (Figs. 9 and 10). In some areas, fibrosis was thicker and more intense, obliterating the surrounding alveoli. The examination also showed areas with a rare honeycomb pattern with no significant interstitial inflammatory infiltration and centriacinar emphysema.

**Fig. 11:**
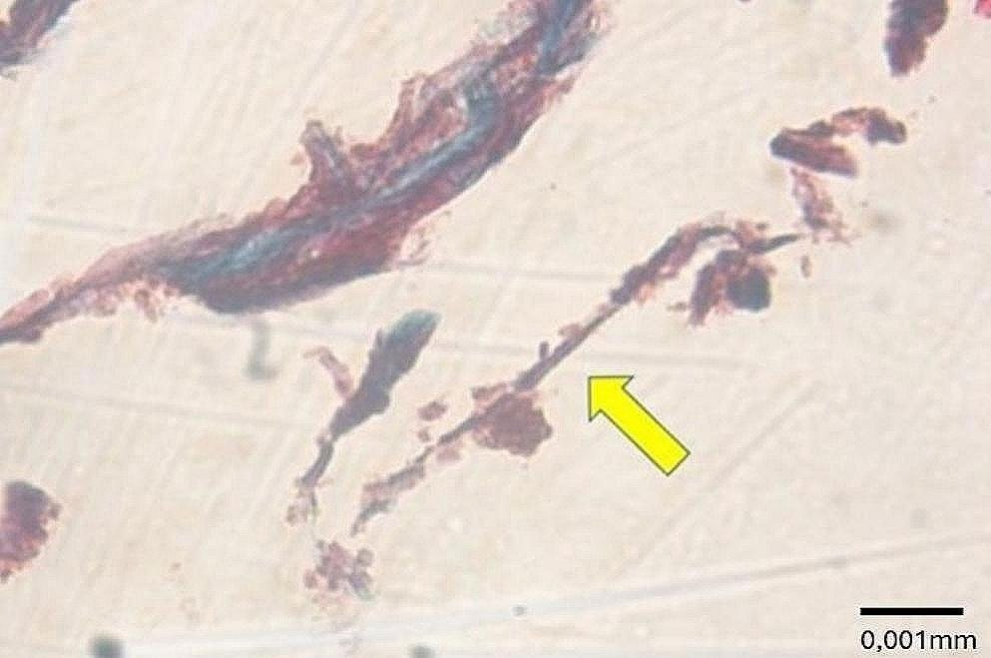
Amorphous fusiform structure compatible with asbestos fiber (*yellow arrow*), located in thealveolar space with fibrotic thickening of the wall. (Masson trichrome, 100X)

Observing samples processed with either hematoxylin-eosin or Perls Prussian blue showed evidence of multiple fusiform structures consistent with the presence of asbestos fibers inside and at a distance from the peribronchial interstitium (Fig. 11).

Pulmonary samples were submitted to an immunochemistry (IHC) investigation, performed using the following antibodies:

-BAP-1 (“**B**RCA1 **A**ssociated **P**rotein**-1**, located on chromosome 3p21.1” for the differentiation of Mesothelioma from benign mesothelial proliferation); calretinin “Calcium-binding protein” for the differentiation (as part of a panel) of pleural Mesothelioma (positive) from lung adenocarcinoma (negative); cytokeratins 5/6 (“Basic (type II) cytokeratins of molecular weight 58 kDa (CK5) and 56 kDa (CK6)”) to distinguish epithelioid Mesothelioma (CK5/6 + in 83%) from lung adenocarcinoma (CK5/6- in 85%); WT-1 (“WT1 gene encodes for Wilms tumor protein located on chromosome 11p13” to differentiate malignant mesothelioma (WT1+) from non small cell lung carcinomas (WT1-); D2-40 “D2-40 Podoplanin is a 40 kDa, transmembrane, oncofetal, O linked sialoglycoprotein (mucin-type) found on lymphatic endothelium, mesothelium, and fetal testis” to differentiate Mesothelioma (+), even in effusions, versus adenocarcinoma (-); HBME-1 (”Marker of mesothelial cells, named after the laboratory of Dr. Hector Battifora and MEsothelioma”) to label mesothelial cells, both benign and malignant (malignant Mesothelioma), and for the identification of Mesothelioma.

The results were negative.

### Scanning electron microscopy (SEM)

**Fig. 12:**
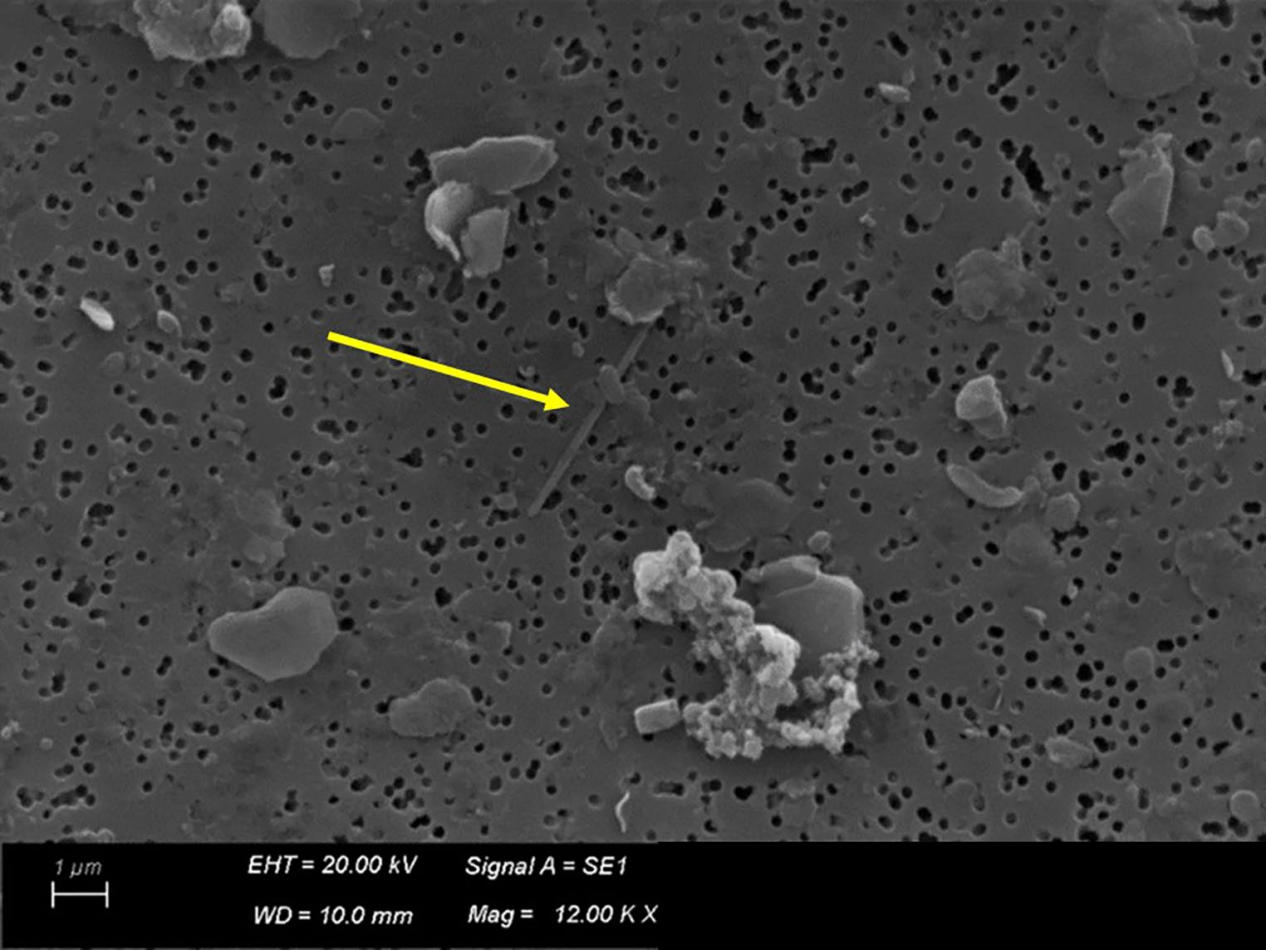
Image of an asbestos fiber (*yellow arrow*) detected during the analysis (magnification 12000X)

Pulmonary samples were examined using SEM for research and identification of asbestos fibers (Fig. 12) as previously described [15]. Analyses were carried out at the Electron Microscopy Center of the Environmental Protection Agency of Lombardy Region (CRME). The results are described in Table 1. The margin of error is expressed as the bounds of the 95% confidence interval. The limit of detection is defined as the bound of the 95% confidence interval of the Poisson distribution and serves as an indicator of the analysis’s sensitivity. The percentage of commercial amphibole asbestos (amosite + crocidolite) is 100%. The average length and diameter of the asbestos fibers are 3.22 microns and 0.22 microns, respectively.

**Table 1:** Results of quantitative analysis of asbestos fibers through scanning electron microscopy (SEM)

Type of fiber	Unit of measurement	Results	Margin of error	Detection limit
Asbestos fibers* Lung	ff/gps (> 1 μm)	13.000.000	8.500.000–18.000.000	320.000
Non-asbestos fibers Lung	ff/gps (> 1 μm)	3.000.000	1.200.000–6.100.000	320.000

*commercial amphibole asbestos fibers (amosite + crocidolite)

Death was attributed to the underlying diseases, given the patient’s age, his medical history (cardiac diseases, old age, loss of weight) an the absence of clinical evidence suggestive of a severe asbestos-related cardiopulmonary disease. Therefore, a causal relationship between the death and the asbestosis was excluded. Asbestosis diagnosis was confirmed based on findings from the the investigations. The diagnosis of mesothelioma was excluded.

## Discussion

The purpose of the investigations was to ascertain the presence of asbestosis and to understand its role in determining death.

The combination of multiple elements acquired through collaboration with the judicial authority and other specialists allowed us to reach the diagnosis relating to death. The death-asbestos correlation was ruled out using both clinical and post-mortem instrumental evidence. Although the existence of asbestosis was confirmed, there were no diagnostic elements suggestive of mesothelioma.

Radiological examinations performed before death and postmortem showed multiple pleural thickenings in the parietal and diaphragmatic regions. It is acknowledged that radiology constitutes an indispensable support in all areas of forensic and insurance medicine [16]. In particular, the literature confirms the presence of pleural plaques (especially in lower-lung regions) as a common manifestation of asbestos-related disease, along with peribronchial fibrosis, subpleural bands, parenchymal lines, and honeycomb pattern [17–19]. The association with previous radiological examinations performed during the man’s life defined a typical picture of asbestosis and excluded the presence of features typical of mesothelioma in imaging.

Regarding the histopathologic findings, pleural plaques represent one of the earliest and most frequent manifestations of asbestos exposure [20] and are described as a common finding at autopsy [21]. In this case, the plaques examined during autopsy and the subsequent histopathological investigations were consistent with asbestos exposure.

Regarding lung fibrosis, the histological samples showed diffuse fibrosis of respiratory bronchiole walls, extending to alveolar ducts and adjacent alveoli, and in some areas, a thicker and more intense fibrosis obliterating the surrounding alveoli. The examination also showed areas with rare honeycomb-pattern changes. This pattern can be classified as a Grade 3 pattern according to the “*Histologic Grading Scheme for Asbestosis*” provided by the Asbestosis Committee of the College of American Pathologists and Pulmonary Pathology Society [10], defined as fibrotic thickening of the walls of all alveoli ≥ 2 adjacent bronchioles. This evidence, in combination with pleural plaques and fibrosis, represents a pathognomonic sign in asbestosis diagnosis.

Furthermore, amorphous fusiform structures compatible with asbestos fibers were found in alveolar spaces. The diagnosis was based on the presence of two or more asbestos fibers per square centimeter of a 5-*µ*m-thick lung Sect. [10]. Still, the date on which we performed the histological examination mut be considered which was 357 days after the man’s death. Indeed, it has been observed that asbestos fiber concentrations decrease after death due to postmortem autolytic phenomena [13]. The IHC analysis was performed using the markers suggested from Italian Guidelines for the Histopathological diagnosis of pleural Mesothelioma [22]. In this regard, a positive result was not returned for the IHC investigation, as degradation of tissue because of postmortem decomposition is known to be a significant limiting factor of IHC [23].

Moreover, no signs of mesothelioma were found. According to Italian national oncology guidelines, the diagnosis of mesothelioma is usually complex and based on a multidisciplinary approach, integrating histopathological examinations, IHC signs and radiological scans [22]. Neither radiological nor histopathologic evidence was found, and, as previously reported, immunochemistry investigation of decomposed tissue has its own limitations, due to degradation of proteins after death. As stated by *the International Mesothelioma Interest Group*, although immunohistochemical stains are essential for confirmation of the diagnosis, “they should not be used to force a tumor into the diagnosis of mesothelioma when it does not look like a mesothelioma on hematoxylin-eosin–stained slides; neither should the stains be performed automatically or blindly without considering several factors” [24]. In the literature, other markers are suggested for the diagnosis of malignant mesothelioma and asbestos exposure, such as specific micro-RNAs and IL-18 [25, 26]. However, given the extended post-mortem interval, application of such markers would not have been useful in the presented case. Lastly, Helsinki criteria recommend the following guidelines: it has to be proven the presence of over 1 million amphibole fibers (> 1 μm) per gram of dry lung tissue as measured by electron microscopy in a qualified laboratory [11]. The analysis of lung asbestos fiber burden through SEM represents a valid support for acquiring knowledge on the nature and entity of asbestos exposure, which is essential in legal medicine evaluation [15], therefore, the use of SEM is crucial for the autopsy diagnosis of asbestos-related diseases. In this regard, our results showed 13.000.000 commercial amphibole asbestos fibers (amosite + crocidolite) > 1 μm/GPS, confirming the high probability of professional asbestos exposure.

It is clear how conducting an autopsy and using standardized multidisciplinary diagnostic algorithms in the postmortem determination of an asbestos-related pathology are essential. The SEM analysis was essential for the diagnosis and it is highly recommended for the detection of asbestos fibers in lung tissue samples [15]. Forensic investigations are dependent on interdisciplinary approaches to reconstruct complex cases and to shed light on the dynamics of the events, especially in severely altered bodies (i.e., when extensively decomposed, mummified, charred, or dismembered) [27–29].

## Conclusion

Presented for this case are the findings of an autopsy performed 357 days after a man’s death following exhumation because of suspected asbestos-related diseases resulting from occupational exposure. We demonstrated the efficacy and reliability of our diagnostic protocol in detecting asbestosis and asbestos fibers and excluding mesothelioma even in decomposed tissues. According to our findings autopsy is still the diagnostic gold standard in the case of suspected asbestosis, in a forensic context even 1 year after death, therefore an autopsy is always highly recommended even in cases where the body have decomposed.

## Key points

1. Autopsy still represents the gold standard for the asbestos-related diseases in forensic context.
2. Even in highly decomposed bodies a multidisciplinary approach based on PMCT, autopsy, histopathology and SEM analysis is reliable to perform a post-mortem asbestosis diagnosis.

## Data Availability

The data presented in this study are available on request from the corresponding author.
